# Exploration and preliminary clinical investigation of an adhesive approach for primary tooth restoration

**DOI:** 10.7555/JBR.36.20220188

**Published:** 2022-11-28

**Authors:** Xiangqin Xu, Jiansheng Zhu, May Lei Mei, Huaying Wu, Kaipeng Xie, Shoulin Wang, Yaming Chen

**Affiliations:** 1 Comprehensive Outpatient Department of Stomatology, the Affiliated Hospital of Stomatology, Nanjing Medical University, Nanjing, Jiangsu 210029, China; 2 Department of Stomatology, Women's Hospital of Nanjing Medical University, Nanjing Maternity and Child Health Care Hospital, Nanjing, Jiangsu 210004, China; 3 School of Public Health, Nanjing Medical University, Nanjing, Jiangsu 211166, China; 4 Department of Child Healthcare, Women's Hospital of Nanjing Medical University, Nanjing Maternity and Child Health Care Hospital, Nanjing, Jiangsu 210004, China; 5 Faculty of Dentistry, University of Otago, Dunedin 628040, New Zealand; 6 Department of Public Health, Women's Hospital of Nanjing Medical University, Nanjing Maternity and Child Health Care Hospital, Nanjing, Jiangsu 210004, China; 7 Jiangsu Key Laboratory of Oral Diseases, Nanjing Medical University, Nanjing, Jiangsu 210029, China

**Keywords:** primary teeth, Single Bond Universal, shear bond strength, marginal microleakage, dentistry, restoration

## Abstract

The current study aims to investigate a suitable adhesive for primary tooth enamel. Shear bond strength (SBS) of primary teeth and the length of resin protrusion were analyzed using one-way ANOVA with Bonferroni multiple comparison tests after etching with 35% H_3_PO_4_. SBS and marginal microleakage tests were conducted with Single Bond Universal (SBU)/Single Bond 2 (SB2) adhesives with or without pre-etching using a nonparametric Kruskal-Wallis test. Clinical investigations were performed to validate the adhesive for primary teeth restoration using Chi-square tests. Results showed that the SBS and length of resin protrusion increased significantly with the etching time. Teeth in the SBU with 35% H_3_PO_4_ pre-etching groups had higher bond strength and lower marginal microleakage than those in the SB2 groups. Mixed fractures were more common in the 35% H_3_PO_4_ etched 30 s + SB2/SBU groups. Clinical investigations showed significant differences between the two groups in cumulative retention rates at the 6-, 12- and 18-month follow-up evaluations, as well as in marginal adaptation, discoloration, and secondary caries at the 12- and 18-month follow-up assessments. Together, pre-etching primary teeth enamel for 30 s before SBU treatment improved clinical composite resin restoration, which can provide a suitable approach for restoration of primary teeth.

## Introduction

During the past few decades, composite resins have been widely used in pediatric dentistry, and the enhanced esthetics required in pediatric dental procedures has increased the use of resin restorations^[1]^. The bonding strength, an important factor for clinical effectiveness of the composite resin restorations, depends largely on the adhesive used during the restoration. However, failures in restoration of primary teeth are still a common problem, with a failure rate varying between 13.6% and 22.5%^[2]^. Thus, it is becoming a challenge to find effective bonding processes for pediatric dentists. Typically, primary and permanent teeth have different microstructures and compositions, which may interfere with clinical performance of adhesive restorations^[3]^. Adhesives for primary teeth are often prepared based on the examination of permanent teeth. In general, the adhesion to primary enamel is not as reliable as that to permanent enamel^[4]^. The acid etching technique creates micropores on the surface of enamel, thus increasing the adherence of composites to the surface. At present, the most commonly used substance of the acid etching technique in clinical settings is 30%–40% phosphoric acid (H_3_PO_4_). The acid-etching process creates a porous enamel surface with a depth of 5 to 50 µm, which allows the penetration of the low-viscosity bonding resin. Formation of resin micro tags within the enamel surface constitutes the fundamental mechanism for resin-enamel adhesion; however, the weakened bonding along the interface may lead to failure.

In pediatric dentistry, there is a great need to shorten the application time, which can reduce the possibility of saliva contamination, especially when treating uncooperative children. A reduction in clinical application steps can shorten chair side time, providing an added advantage in treating pediatric patients. The recent trend of quickening clinical application steps advocates the reduction of application times in pediatric dentistry, and the use of self-etching adhesives seems to be an attractive choice. At present, the choice of etch-and-rinse (E&R) or self-etching (SE) adhesive systems remains controversial in pediatric dentistry. To our knowledge, SE adhesives enable a shorter operation time, thus providing an advantage in treating pediatric patients. However, some studies found that the SE adhesive system provided insufficient adhesion to primary teeth^[5–6]^, while other studies showed more marginal leakage with the SE adhesive system on primary teeth^[7]^. E&R adhesive systems have both advantages, such as higher adhesion for restoring primary teeth, and disadvantages, such as a long clinical operation time and a high technical sensitivity. Thus, some studies proposed that pre-etching enamel before using SE adhesives could significantly increase the bonding strength.

There is little information in the literature on the performance of universal adhesive systems that applied to primary teeth with different bonding strategies. Some dentisits have used additional enamel etching to improve the insufficient bonding strength, but it remains controversial whether additional etching increases the bond strength of one-step SE adhesives that have been developed recently. In addition, polymerization shrinkage of the composite can lead to micro-gap formation, microleakage, recurrent caries, and partial or complete restoration replacement. Many scholars have compared the edge sealing properties of SE and E&R adhesive systems, but no unified conclusion has been reached^[8–9]^. Above all, the controversies, which regard the therapeutic standard for primary teeth, confirm persistent lack of the reference scientific studies.

Therefore, the present study aims to identify an effective approach for primary tooth bonding. We first investigated the effects of the etching time with 35% H_3_PO_4_, an optimal adhesive system and the mode used for bonding to primary teeth *in vitro*. Then, we performed a longer clinical observation (up to 18 months) of resin composite restorations to better understand the material's performance in the oral cavity to provide an experimental basis for clinical application.

## Materials and methods

### Sample preparation

Primary teeth, which were extracted due to retention for future use, were collected from patients aged between five and 12 years at the Department of Stomatology of Nanjing Maternity and Child Health Care Hospital. Teeth with caries, restorations, enamel defects, and any congenital anomalies were excluded. After cleaning and removal of the connective tissue, teeth were immersed in 0.5% chloramine solution at 4 ℃ for up to one month^[10]^. All teeth were washed with the filtered phosphate-buffered saline (PBS, pH 7.4) and stored in a saline solution that was replaced every week. The present study was approved by the Ethical Committee of Nanjing Maternal and Child Health Hospital (Approval No. 2020-KY-019), and all the participants signed an informed consent form before participating in the study.

### Determination of the shear bond strength of primary teeth etched in 35% H_3_PO_4_ for different periods

For quantitative analysis, shear bond strength (SBS) is a major outcome in this study, and thus we expected to detect a difference of at least 2 MPa. Considering that the common standard deviation is 1.5 MPa with a power of 0.80, the sample size should be at least nine pieces per group, and we set the sample size as 10 pieces per group. Ten primary molars were collected, and each tooth was cut into four pieces with a cross-shaped incision along the crown surface using a low-speed cutting machine (Isomet, Buehler Ltd., Lake Bluff, IL, USA). The four pieces were randomly divided into groups to be exposed to 35% H_3_PO_4_ (Bisco Inc., Schaumburg, IL, USA) with different etching times (0, 15, 30, and 60 s). Each piece was embedded with the exposed buccal surface, and then ground and polished with 600 grit silicon carbide paper (Polimet, Buehler Ltd.) to obtain flat enamel. The adhesive agent (Adper Single Bond 2 [SB2], 3 M/ESPE, St. Paul, MN, USA) was applied to the etched enamel surfaces following the manufacturer's instructions. A Teflon mould with a height of 2 mm and diameter of 1.5 mm was placed on the enamel surfaces. A customized fixture was used to hold the mould. Composite resin (FiltekZ350, 3 M/ESPE) was applied, and each layer was cured for 40 s, and then the specimens were stored in the distilled water for 24 h. The SBS test was performed using a universal testing machine (Instron 3365, Instron Co., Norwood, MA, USA). A shear force was applied perpendicularly to the resin cylindrical button at a distance of 1 mm from the enamel surface to the loading head. The loading speed was set to 0.5 mm/min until the resin and tooth surface broke^[11]^, and the experimental data were recorded. The load necessary to debond the resin composite was recorded in Newtons (N). The bond strength was expressed in MPa by dividing the load at failure by the bonded surface area measured in mm^2^. All data were entered into the calculation formula, and the shear bonding strength was calculated in turn. The calculation formula is as follows: *P*=*F*/*S*, where *P* is shear bonding strength, MPa; *F* is maximum shear force value when the filling body fractures, N; and *S* is the bonding area, mm^2^.

### Observation of the etched enamel surface and the resin protrusion of primary teeth under scanning electron microscopy

Ten primary molars were collected, and two were cut into four pieces and etched for SEM observation as described in the previous section. Another eight primary molars were restored with resin after etching, and the process was the same as that in the SBS test. After incubation in the distilled water for 24 h, the specimens were cross-sectioned. Resin tag analysis was performed by measuring the depth of two petals of the specimen resin tags under scanning electron microscopy (SEM). Four areas were randomly selected from each piece for assessment of the resin tag length; in total, 16 data points were obtained from each group, and the average of each photograph was calculated^[12]^. All the specimens prepared for SEM were dehydrated, dried step by step in an aqueous ethanol solution, and then sputter-coated with gold for observation^[13]^. An accelerating voltage of 10.0 kV (Tescan, Maia 3, xmu, Czech Republic) was used.

### Determination of the shear bond strength of primary teeth in different adhesive methods and fracture modes

Fifteen primary molars were collected and cut as described in the previous section, and all samples were randomly assigned into six groups (*n*=10 for each group). The samples were randomly grouped by the etching time (0, 15, and 30 s) and adhesive agent (SB2/Single Bond Universal (SBU), 3 M/ESPE, St. Paul, MN, USA) as follows: control, SBU, 35% H_3_PO_4_ 15 s + SB2, 35% H_3_PO_4_ 15 s + SBU, 35% H_3_PO_4_ 30 s + SB2, and 35% H_3_PO_4_ 30 s + SBU. The samples were embedded and restored with resin for the SBS test. After the SBS test, the fractured test specimen surfaces were examined visually under a stereoscopic loupe (SMZ1500, Nikon, Kanagawa, Japan) at 40× magnification. The fracture types were classified as follows: adhesive failure (failure between the adhesive and enamel), cohesive failure in the enamel, cohesive failure in the resin, and mixed failure (adhesive and cohesive failure in the resin).

### Detection of the marginal microleakage of primary teeth in different adhesive methods

Sixty primary incisors were randomly divided into six groups (*n*=10). The roots were embedded, and a cavity with a diameter of 2 mm and a depth of 1.5 mm was prepared in the central labial surface of the crown. Then, the etching and resin bonding were performed, and the teeth were kept in a 37 ℃ water bath for 24 h. The teeth were placed in a thermal cycler (THE-1100, SD Mechatronik, Feldkirchen-Westerham, Germany) for 5000 cycles. Two layers of nail polish were spread evenly over approximately 1 mm around the bonding surface. The teeth were soaked in a 0.5% alkaline fuchsin solution for 24 h and then removed and cleaned. The teeth were cut vertically through the centre of the filling, exposing the bonding surface. Staining of the tooth surface was observed at 40× magnification (SMZ1500, Nikon) to evaluate the level of microleakage according to the depth of dye penetration. Marginal microleakage was evaluated with grades 0 to 4^[14]^.

### Clinical observation of resin restoration of Single Bond Universal for primary teeth preetched with or without 35% H_3_PO_4_

A total of 42 patients with 84 carious teeth who were treated in the Stomatology Department of Nanjing Maternal and Child Health Hospital between August 2020 and August 2021 were selected (19 males and 23 females) with a mean age of 5.5 years. All patients had two primary molars with moderate Class Ⅱ cavities simultaneously in the same quadrant of the mouth, and the caries inspection standard was based on the American Dental Association (ADA) dental caries diagnostic criteria. Patients with rampant caries, severe periodontitis or poor oral hygiene, pathological wear and bad chewing habits were excluded. We obtained an informed consent from both the patients and their parents, and assured of a smooth postoperative follow-up. Two molars were randomly divided into the experimental group (SBU with 35% H_3_PO_4_ pre-etching for 30 s) and the control group (SBU). Patients were provided with oral hygiene instructions preoperatively. A rubber dam was placed on all patients before restoration, the teeth were cleaned and dried, and adhesive agent (Single Bond Universal, 3M/ESPE) and composite resin (FiltekZ350, 3M/ESPE) were applied according to the manufacturers' recommendations. Prior to the adhesive procedures, the selective etching was performed on the enamel with 35% H_3_PO_4_ in the experimental group. Restorations were finished and polished immediately after placement. All lesions were restored by the same dentist. All restorations were scored with regard to retention, marginal discoloration, marginal adaptation, sensitivity, and secondary caries using modified United States Public Health Service (USPHS) criteria^[15]^; this was done one week after placement (baseline), and at six, 12, and 18 months.

### Statistical analysis

All analyses were performed with GraphPad Prism Pro 5.0 (GraphPad Software, Inc., San Diego, CA, USA) and IBM-SPSS 25 (IBM Corp., Armonk, NY, USA). Descriptive statistics of numbers and percentages were used to describe the samples. A normal distribution of the data was assessed using the Shapiro-Wilk test for normality. One-way ANOVA with Bonferroni multiple comparison tests was used to compare the SBS and resin tag length across the different etching time groups. A nonparametric Kruskal-Wallis test was used to detect the differences in marginal microleakage levels. Statistical evaluations of the clinical data were performed using Chi-square tests or Fisher's exact test. Statistical significance was set at *P*<0.05.

## Results

### Effects of the etching time with 35% H_3_PO_4 _on the etched pattern and shear bond strength of primary teeth

**Figure 1:**
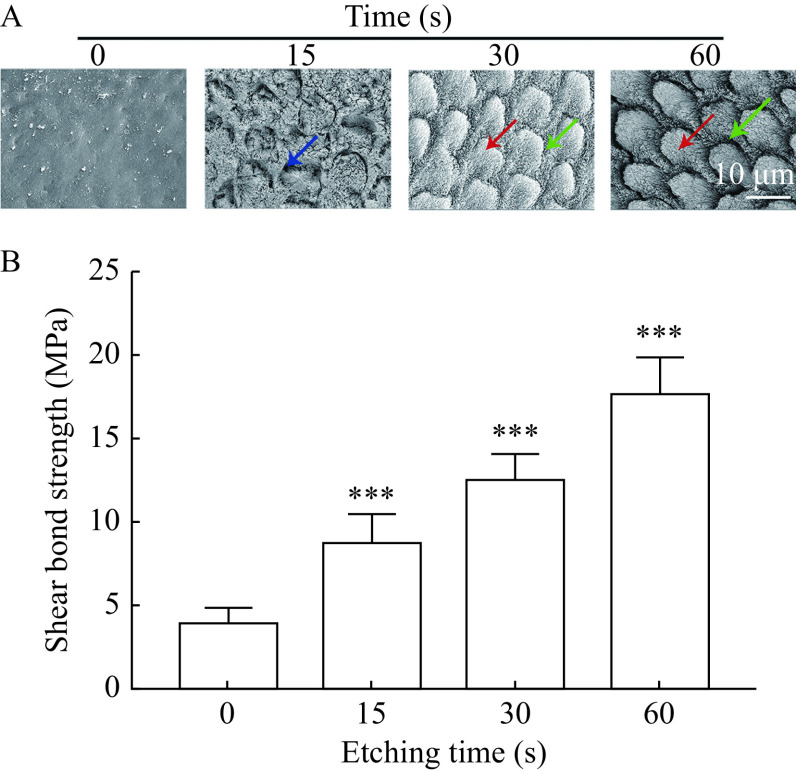
Effects of the etching time of phosphoric acid on the enamel surface and shear bond strength of primary teeth.

The analysis of SEM micrographs revealed that when etched for 15 s, the enamel surface showed an uneven structure; the interprismatic matrices dissolved first, exposing the enamel prism head; and some areas showed the type Ⅰ etching pattern. When conditioned for 30 and 60 s, the surface topography exhibited a uniform honeycomb pattern, which is the type Ⅱ etching pattern (***Fig. 1A***). One-way ANOVA indicated a significant effect (*P*<0.001) of the etching time on SBS, and the results showed that there were significant differences among the groups with different etching times (*P*<0.001). As the etching time increased, the SBS increased significantly (*P*<0.001) (***Fig. 1B***)*.*

### Effects of the etching time with H_3_PO_4 _on the length of resin penetration of primary teeth

**Figure 2:**
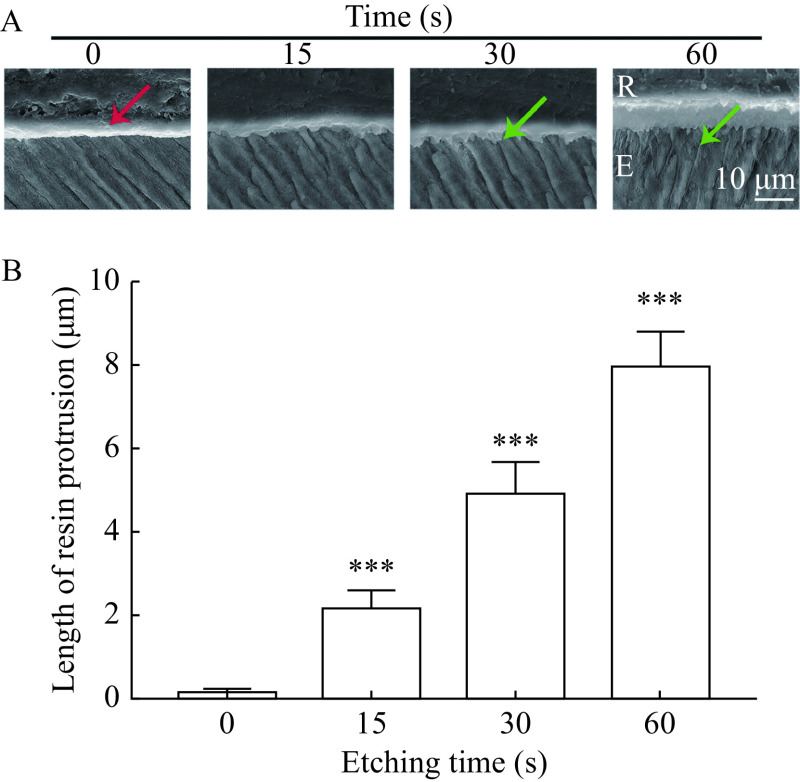
Effects of the etching time of phosphoric acid on the length of resin protrusion of primary teeth.

SEM observations revealed an authentic hybrid layer with a distinct resin-enamel interdiffusion zone, in which a high density of long resin protrusions could be noted (***Fig. 2A***). The resin protrusions were uneven in length and distributed in clusters. As shown in ***Fig. 2B***, the length of resin protrusion significantly increased with the etching time (*P*<0.001).

### Effects of different adhesive methods on the shear bond strength of primary teeth

**Figure 3:**
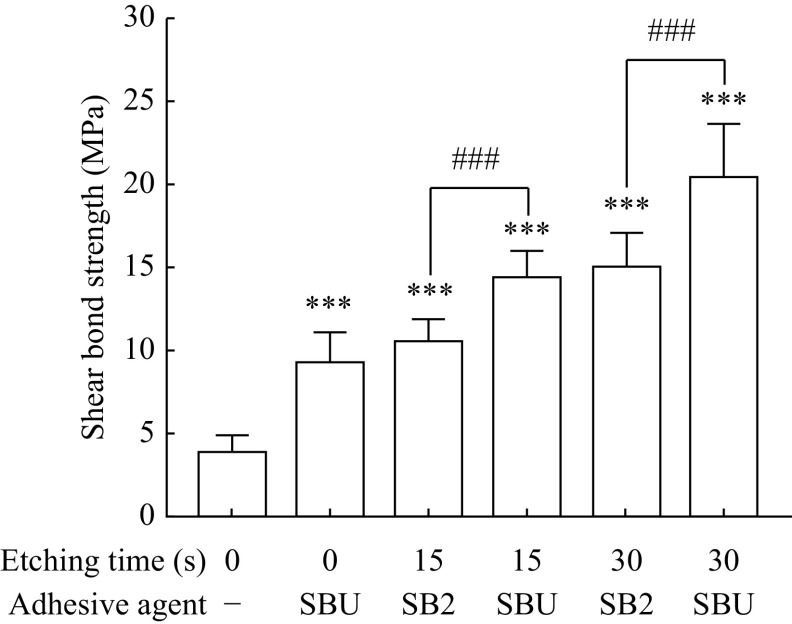
Effects of different adhesive methods on the shear bond strength of primary teeth.

The SBS in different adhesive methods was analyzed. The teeth in the 35% H_3_PO_4_ etched 30 s + SBU group had the highest SBS at (20.40±3.24) MPa. The SBS of treatment groups were significantly higher than that of the control. The teeth in the 35% H_3_PO_4_ etched with SBU groups had significantly higher SBS than those in the SB2 group that were etched for the same amount of time (*P*<0.001) (***Fig. 3***). The SBS was mainly related to the different acid-etching times and adhesive systems.

### Effects of different adhesive methods on the fracture mode of primary teeth

**Figure 4:**
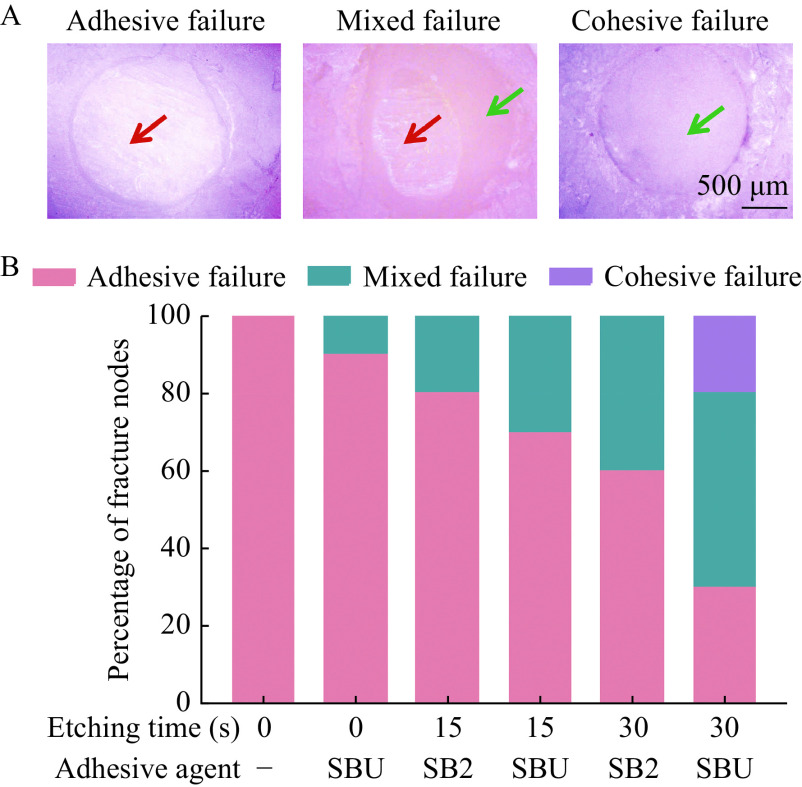
Effects of different adhesive methods on the fracture mode.

The 35% H_3_PO_4_ etched 30 s + SBU group had two specimens that appeared cohesive in resin fracture, and these specimens were assigned to the group of mixed fractures, when the results were analyzed (***Fig. 4A***). Most specimens showed adhesive fractures, and as the SBS increased, more mixed fractures appeared (0, 10%, 20%, 30%, 40%, and 50%) (***Fig. 4B***). The appearance of mixed and cohesive fractures indicated an increase in the strength of the bond interface or adhesive. Compared to teeth in the control group, there were more mixed fractures in teeth in the 35% H_3_PO_4_ etched 30 s + SB2/SBU groups (*P*<0.05) (***Table 1***), indicating a firmer bond of the exposed materials.

**Table 1:** Distribution of the fracture modes among different groups

Groups	Fracture modes, *n* (%)			*P-*value
	Adhesive failure	Mixed failure	Cohesive failure	
Control	10 (100)	0	0	
SBU	9 (90)	1 (10)	0	0.136^a^
35% H_3_PO_4_ 15 s + SB2	8 (80)	2 (20)	0	0.305^a^
35% H_3_PO_4_ 15 s + SBU	7 (70)	3 (30)	0	0.060^a^, 0.264^b^
35% H_3_PO_4_ 30 s + SB2	6 (60)	4 (40)	0	**0.025** ^a^
35% H_3_PO_4_ 30 s + SBU	3 (30)	5 (50)	2 (20)	**0.005**^a^, 0.211^c^
Fisher's exact test was used to compare the differences in treatment outcomes. ^a^Compared with the control group; ^b^compared with the 35% H_3_PO_4_ 15 s + SB2 group; ^c^compared with the 35% H_3_PO_4_ 30 s + SB2 group. Bold values indicate *P*<0.05. SBU: Single Bond Universal; SB2: Single Bond 2.				

### Effects of different adhesive methods on the marginal microleakage of primary teeth

**Figure 5:**
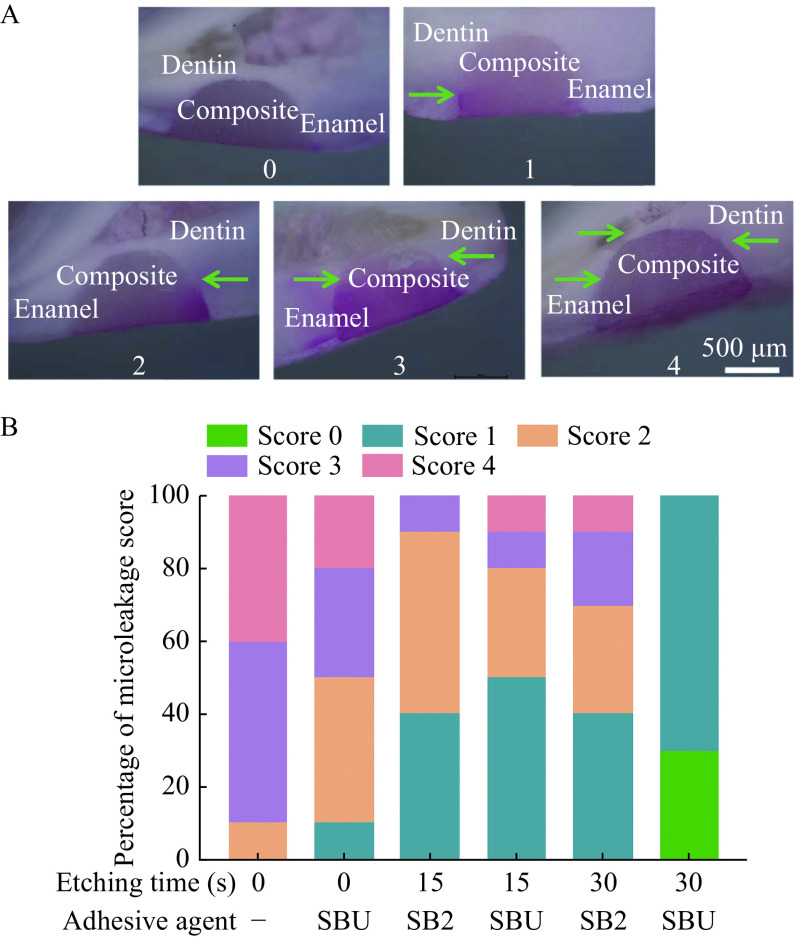
Effects of different adhesive methods on the microleakage of the resin of primary teeth.

Typical representative diagrams of microleakage are shown in ***Fig. 5A***. Stacked bar charts illustrate the relative abundance of marginal microleakage scores across different groups. The marginal microleakage scores of the experimental groups were reduced (***Fig. 5B***, ***Table 2***). There were significant differences in the experimental groups, except for the SBU group (*P*<0.05). In addition, there was a significant difference between the 35% H_3_PO_4_ 30 s + SBU group and the 35% H_3_PO_4_ 30 s + SB2 group (***Table 2***).

**Table 2:** Comparison of the microleakage grade scores among different groups

Groups	Microleakage score, *n* (%)					*P*-value
	0	1	2	3	4	
Control	0	0	1 (10)	5 (50)	4 (40)	
SBU	0	1 (10)	4 (40)	3 (30)	2 (20)	0.265^a^
35% H_3_PO_4_ 15 s + SB2	0	4 (40)	5 (50)	1 (10)	0	**0.004** ^a^
35% H_3_PO_4_ 15 s + SBU	0	5 (50)	3 (30)	1 (10)	1 (10)	**0.015** ^a^
35% H_3_PO_4_ 30 s + SB2	0	4 (40)	3 (30)	2 (20)	1 (10)	**0.020**^a^
35% H_3_PO_4_ 30 s + SBU	3 (30)	7 (70)	0	0	0	**0.001**^a^, **0.044**^b^
Fisher's exact test was used to compare the differences in treatment outcomes. ^a^Compared with the control group; ^b^compared with the 35% H_3_PO_4_ 30 s + SB2 group. Bold values indicate *P*<0.05. SBU: Single Bond Universal; SB2: Single Bond 2.						

### Clinical effects of resin restoration with Single Bond Universal for primary teeth preetched with or without 35% H_3_PO_4_

During the 6-, 12- and 18-month follow-up assessments, the intact retention rates for restorations of the SBU with pre-etching 30 s group showed slight decreases of 97.62%, 92.86%, and 90.47%, respectively. In the SBU group, a statistically significant change was observed for the 12-month follow-up assessment, and only 71.42% of the restoration was maintained during the 18-month follow-up assessment. Furthermore, there were significant differences in the restoration retention rates between the two groups at the 6-, 12-, and 18-month follow-up assessments (*P*<0.05).

As shown in ***Table 3***, 2 (4.76%), 8 (19.5%), and 10 (23.81%) restorations in the SBU group showed total cracks in marginal adaptation during the 6-, 12-, and 18-month follow-up assessments. Significant differences were observed at 12 and 18 months (*P<*0.05) between the two groups. Superficial discoloration appeared at the six-month follow-up assessment, and the marginal discoloration rate increased to 23.81% over 18 months in the SBU group. In the pre-etching group, discoloration appeared at the 12-month follow-up assessment, with a slight increase at 18 months (9.52%). There was a significant difference between the two groups at 12 and 18 months (*P*<0.05).

**Table 3:** Clinical evaluation of resin restoration with SBU for primary teeth preetched with or without 35% H_3_PO_4 _at 6, 12, and 18 months

Evaluation criteria	6 months, *n* (%)			12 months, *n* (%)			18 months, *n* (%)	
	SBU (*N*=42)	Preetch (*N*=42)		SBU (*N*=42)	Preetch (*N*=42)		SBU (*N*=42)	Preetch (*N*=42)
Retention								
Partial loss	3 (7.14)	1 (2.38)^*^		6 (14.29)	2 (4.76)^**^		8 (19.05)	2 (4.76)^**^
Completely loss	1 (2.38)	0 (0.00)		3 (7.14)	1 (2.38)^*^		4 (9.52)	2 (4.76)^*^
Total	4 (9.52)	1 (2.38)^*^		9 (21.43)	3 (7.14)^**^		12 (28.57)	4 (9.52)^**^
Marginal adaptation								
Partial crack	2 (4.76)	1 (2.38)		5 (11.90)	2 (4.76)^*^		6 (14.28)	3 (7.14)^*^
Obvious cracks	0 (0.00)	0 (0.00)		3 (7.14)	1 (2.38)^*^		4 (9.52)	2 (4.76)^*^
Total	2 (4.76)	1 (2.38)		8 (19.05)	3 (7.14)^*^		10 (23.81)	4 (9.52)^*^
Marginal discoloration								
Discoloration	2 (4.76)	0 (0.00)		4 (9.52)	1 (2.38)^*^		6 (14.28)	2 (4.76)^*^
Visibly discoloration	0 (0.00)	0 (0.00)		2 (4.76)	1 (2.38)		4 (9.52)	2 (4.76)^*^
Total	2 (4.76)	0 (0.00)		6 (14.28)	2 (4.76)^*^		10 (14.28)	4 (9.52)^*^
Secondary caries	1 (2.38)	0 (0.00)		4 (9.52)	1 (2.38)^*^		6 (14.28)	2 (4.76)^*^
Chi-square test was used to compare the pre-etching group and SBU group. ^*^P<0.05, ^**^P<0.01. SBU: Single Bond Universal.								

One case of secondary caries was detected as early as six months (2.38%) in the SBU group, followed by four (9.52%) cases at 12 months, and six (14.28%) cases at 18 months. In the pre-etching group, we detected one and second secondary caries at the 12-month and 18-month follow-up assessments, respectively. There were significant differences between the two groups at the 12- and 18-month follow-up assessments (*P*<0.05).

In general, there were significant differences between groups at 12 and 18 months (*P*<0.05). Additionally, no difference was seen in sensitivity to cold between the two groups.

## Discussion

Acid etching changes the permeability of the enamel surface, making it more conducive to wetting, spreading, and penetrating of the bonding resin^[16]^. Several studies comparing the bond strength to primary and permanent dentine produced varying results^[17–18]^. However, most studies found that primary teeth needed a longer etching time than permanent teeth^[19–20]^, which, apart from their different microstructure and composition, may also be related to a thicker roadless enamel on the primary enamel surface^[21]^, this primeless layer has a uniform crystallite structure that may result in the formation of fewer resin tags.

Our SEM study showed that the morphology of the primary enamel etched surface gradually became uniform within 60 s. Some researchers indicated that in addition to good enamel etching morphology^[22]^, exposing more enamel crystal structures and forming a porous enamel structure were more important than other factors^[23]^. Moreover, we measured the length of the resinous protrusion under SEM. The results of the SEM analysis provided supportive evidence that the enamel achieved a more defined etching pattern as the etching time increased, and these morphological features could be translated into longer resin tags and higher SBS values, which agreed with the findings of a previous study^[24]^.

To resist the contraction forces induced by composite polymerization, it was suggested that the bonding agents used on teeth should achieve an adhesive strength of approximately 17 to 20 MPa^[25]^. The SBS etching for 60 s group presented values within this range. However, in pediatric patients, etching for 60 s is hard to achieve, because both risk of mucosal damage and dentin sensitivity may increase simultaneously. Considering that pediatric dentistry is predominantly affected by patient compliance, the self-etching adhesive system has great advantages in treating pediatric patients. However, our *in vitro* results confirmed that adhesion to primary teeth with this system was insufficient. The SBU adhesive contains the functional monomer methacryloyloxydecyl dihydrogen phosphate (MDP), which enhances bonding through chemical adhesion to the tooth^[26]^. Our clinical investigation found that the restoration retention rate decreased significantly with the follow-up time, which may be due to the facts that mild pH of universal adhesives can only provide inadequate enamel bonding, and the self-etching adhesive system is more prone to fatigue^[27]^.

The purpose of our study was to improve the bond strength while reducing the surface treatment time. We compared the pre-etching (*i.e.*, 15 and 30 s) before the SE adhesive system with the E&R adhesive system, which had been proven to have insufficient adhesion in our study. We found that SBU with pre-etching for 30 s resulted in adhesion close to etching for 60 s in the E&R adhesive system group. In our clinical study, this approach of SUB with pre-etching for 30 s demonstrated a high success rate after 18 months. The retention rate of the restorations showed only a slight decrease (from 97.62% to 90.47%) within 18 months of clinical service, supporting the *in vitro* data on bond strength. The observed outcome may be attributed to the fact that the phosphoric acid pretreatment removed the barrier effect of the smear layer, which was more conducive to the penetration of the bonding monomer of the acid-etching adhesive to the surface of the demineralized enamel^[28]^. In addition, enamel etching increased the bonding ability of MDP monomers^ [29]^. However, enamel pretreatment^[30]^, contamination^[31]^, light exposure^[32]^, and curing time^[33]^ have been demonstrated to significantly influence the bond strength in orthodontics. These variables should be evaluated in future studies.

Marginal microleakage remains a major cause of composite restoration failure despite improvements in restorative materials^[34]^. Studies have reported that the E&R adhesive system is more effective in reducing microleakage^[35–36]^. Our *in vitro* study showed that marginal microleakage in the SBU group was significantly higher than that in the other experimental groups. Consistent with the clinical results, the pre-etching 30 s group had the least microleakage. The USPHS criteria are the most common standards for the clinical evaluation of restorations. Some studies have concluded that there is a certain correlation between marginal adaptation and marginal discoloration. Marginal adaptation is an essential factor for the longevity of restoration, and a poor marginal adaptation may lead to discoloration, sensitivity, and secondary caries. Some clinical studies reported an inferior marginal discoloration or adaptation of universal adhesives over time. In our clinical study, marginal adaptation, marginal discoloration, and secondary caries in the SBU group showed significant changes during the follow-up periods of both 12 and 18 months, and there were significant differences between the two groups at the 12- and 18-month follow-up. Studies have shown that the main reason for the failure of the resin bonding repair was secondary caries^[37]^. There were only two cases of secondary caries in the pre-etching group, which is clinically acceptable. In the present study, only moderate Class Ⅱ cavities were included because of the observed low failure rate of Class Ⅰ restorations. A recent study found that the success rate for Class Ⅱ in primary teeth was 68% at 18 months^[38]^. Based on the American Dental Association criteria, some studies reported that the failure rates due to retention and microleakage must be less than 10% at 18 months^[39]^. Our clinical study found that the success rate of the SBU with pre-etching 30 s group met this criterion. Nevertheless, in the present study, the participants reported no postoperative sensitivity during any evaluation period, which are in agreement with previous studies showing that the use of the selective enamel etching mode resulted in high survival rates^[40–41]^.

In addition to acid etchants, there are alternative techniques for treatment. Laser can roughen the enamel surface and increase the acid resistance of the tooth, which may be a good substitute for acid etching^[42–43]^. But the irradiation distance and the power set in the laser application may increase the difficulty of laser operation and the uncertainty of the results. Air-abrasion technology, as an alternative to the etching technique, can significantly increase the bonding strength^[44–45]^, effectively etch the tooth tissue, and avoid damage to surrounding tissue, yet it has many operational and experimental requirements. At present, the development direction of the bonding system is to simplify the operation steps and overcome the shortcomings of the existing bonding system. Therefore, it is necessary to study new acid etching technologies in the future, and the self-etching adhesive system will still be the focus of future research^[46]^.

Although the SBU simplifies clinical operation, it may cause insufficient adhesion and microleakage of primary tooth enamel. Pretreatment 30 s with 35% H_3_PO_4_ before SBU may improve the bond strength and clinical success of primary teeth. Therefore, further studies should be performed to confirm the results of the present study and to evaluate the impact of other types of universal adhesives. Moreover, studies are also needed to determine the long-term effect of these treatments on bond strength of dentin.

### Conclusions

To summarize, based on our results, SBU with 35% phosphoric acid pre-etching for 30 s was ideal for primary tooth adhesive. The present study confirmed that the SBU without pre-etching was not suitable for primary teeth. Therefore, further clinical studies should be performed to investigate the performance of other types of self-etching adhesive systems. Moreover, longer-term clinical studies are necessary to verify the clinical performance of SBU with pre-etching.
